# Experimental Study Regarding the Behavior at Different pH of Two Types of Co-Cr Alloys Used for Prosthetic Restorations

**DOI:** 10.3390/ma14164635

**Published:** 2021-08-17

**Authors:** Farah Bechir, Simona Maria Bataga, Elena Ungureanu, Diana Maria Vranceanu, Mariana Pacurar, Edwin Sever Bechir, Cosmin Mihai Cotrut

**Affiliations:** 1 Faculty of Dental Medicine, George Emil Palade University of Medicine, Pharmacy, Science, and Technology of Targu Mures, 38 Gh. Marinescu Str., 540142 Targu Mures, Romania; farah.bechir@yahoo.com (F.B.); marianapac@yahoo.com (M.P.); 2 Faculty of Medicine, George Emil Palade University of Medicine, Pharmacy, Science, and Technology of Targu Mures, 38 Gh. Marinescu Str., 540142 Targu Mures, Romania; simonabataga@yahoo.com; 3 Faculty of Materials Science and Engineering, Politehnica University of Bucharest, 313 Splaiul Independentei, 060042 Bucharest, Romania; ungureanu.elena14@yahoo.com (E.U.); diana.vranceanu@upb.ro (D.M.V.); cosmin.cotrut@upb.ro (C.M.C.)

**Keywords:** casting, CAD-CAM milling, salivary pH, Co-Cr dental alloys, corrosion

## Abstract

Cobalt-chromium (Co-Cr) alloys are widely utilized in dentistry. The salivary pH is a significant factor, which affects the characteristics and the behavior of dental alloys through corrosion. This study aimed to evaluate the corrosion behavior in artificial saliva with different pH values (3, 5.7, and 7.6) of two commercial Co-Cr dental alloys manufactured by casting and by milling. Corrosion resistance was determined by the polarization resistance technique, and the tests were carried out at 37 ± 1 °C, in Carter Brugirard artificial saliva. After the electrochemical parameters, it can be stated that the cast Co-Cr alloy has the lowest corrosion current density, the highest polarization resistance, and the lowest speed of corrosion in artificial saliva with pH = 7.6. In the case of milled Co-Cr alloy, the same behavior was observed, but in artificial saliva with pH = 5.7, it recorded the most electropositive values of open circuit potential and corrosion potential. Although both cast and milled Co-Cr alloys presented a poorer corrosion resistance in artificial saliva with a more acidic pH value, the milled Co-Cr alloy had better corrosion behavior, making this alloy a better option for the prosthetic treatment of patients suffering from GERD.

## 1. Introduction

A wide variety of dental alloys for prosthetic restorations are currently traded. The American Dental Association (ADA) classification divides dental alloys into high precious, precious, and non-precious alloys [1,2,3].

Cobalt-chromium (Co-Cr) alloys are among the widely utilized alloys in dentistry and are alternatives to other types of dental alloys used to manufacture fixed prosthetic restorations [4,5]. The castability, the metallic luster, and the corrosion resistance of the dental alloys are important factors for the biocompatibility and feasibility of dental prosthetic restorations [6].

The oral cavity, which has specific individualized conditions, is characterized by its environment having a wide variety of pH, bacterial load, and/or temperature variation [7]. Temperature and pH are two significant factors that affect the electrochemical behavior of dental materials [8].

The affection represented by gastroesophageal reflux disease (GERD) is an accustomed condition [9,10,11,12]. According to some studies published by Patel et al., respectively Antunes et al., GERD affects about 10–20% of the western adults [13,14]. Moreover, according to Eusebi et al., GERD symptoms present a higher estimated prevalence in women than men [15,16]. The effects of GERD are mainly localized in the esophagus (as heartburn, regurgitation, dysphagia, belching, epigastric pain, etc.) [9,14].

Oral symptoms like dental erosion, inflammation, and affections of the oral mucosa, xerostomia, ulceration, and angular cheilitis have also been frequently presented in patients with GERD [17,18,19,20].

In GERD, the pH of the oral fluids is changed to acidic [14], and the phenomenon of pH values reduction in the oral environment influences the characteristics, properties, and behavior of dental materials, including that of dental alloys [21,22].

Thus, the present study aims to evaluate the corrosion behavior in artificial saliva with different pH values (3, 5.7, and 7.6) of two commercial Co-Cr dental alloys used for the manufacturing of prosthetic restorations by two different technologies, namely by casting and milling.

## 2. Materials and Methods

The selected Co-Cr dental alloys were purchased from Simex, Italy (Biomate-K Special) and Scheftner Dental Alloys, Germany (Starbond Esy Disc), and did not contain nickel and beryllium. Nevertheless, Biomate-K Special is indicated by the manufacturer for the casting of metal components of metal-ceramic crowns and bridges. At the same time, Starbond Esy Disc is suitable for milling crowns and dental bridges, metal components of metal-ceramic crowns and bridges, bars, and milled slides, respectively, for superstructures on implants.

The chemical composition specified by the manufacturer of Co-Cr alloys used in the study, along with sample codification, are shown in Table 1.

The shape and dimensions of the samples used in this study were dictated by the type of characterizations and tests performed and by the equipment used. The samples consisted of discs with a diameter of 15 mm and a thickness of 5 mm. They were obtained by casting and milling, in the same dental laboratory, according to the specific technological manufacturing phases of the cast and milled prosthetic restorations.

**Figure 1:**
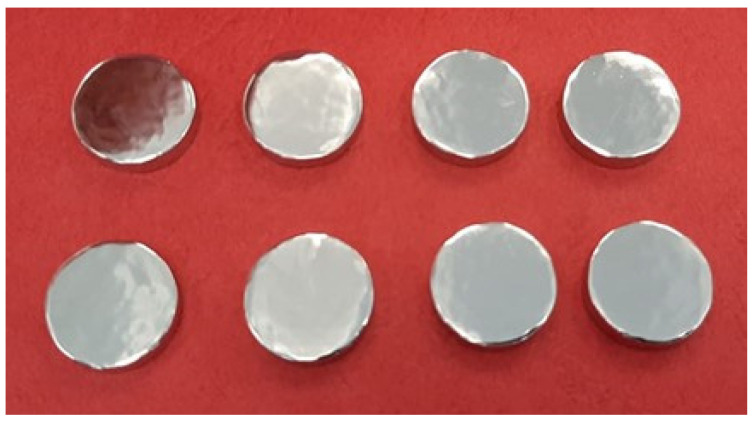
Macroscopic images of the samples obtained from the two types of Co-Cr alloys.

In Figure 1, the macroscopic images of the samples obtained in the dental laboratory after finishing and polishing are presented.

The morphology and elemental composition of the Co-Cr alloy manufactured by casting and/or milling were analyzed with a scanning electron microscope, equipped with an X-ray energy dispersive spectrometer (SEM-EDS, Phenom ProX, Phenom World, Eindhoven, The Netherlands).

Corrosion resistance tests were carried out with a PARSTAT 4000 Potentiostat/Galvanostat (Princeton Applied Research, Oak Ridge, TN, USA). Electrochemical tests were performed according to ASTM G5–94 (2011) by using the linear polarization technique.

## 3. Results

### 3.1. Characterization of Co-Cr Alloys

#### 3.1.1. Morphology

**Figure 2:**
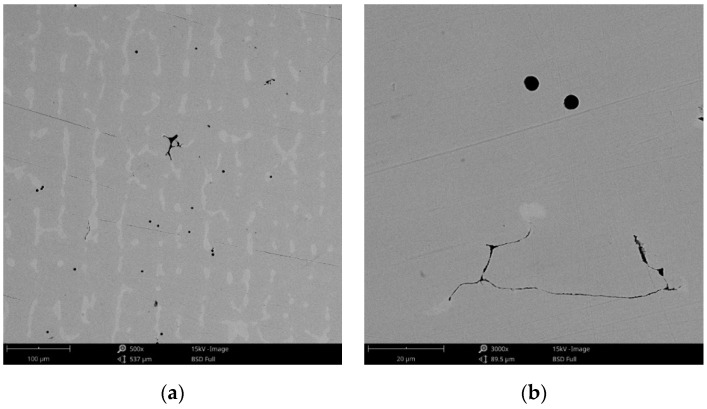
SEM images of the CCc (cast Co-Cr alloy) sample surface at: (**a**) 500×; (**b**) 3000×.

**Figure 3:**
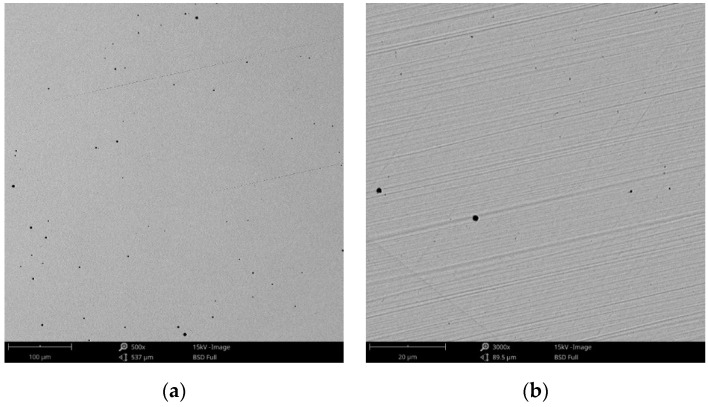
SEM images of the CCm (milled Co-Cr alloy) sample surface at: (**a**) 500×; (**b**) 3000×.

The surface morphology obtained by SEM of the two Co-Cr alloys used in the study, CCc and CCm manufactured by casting and milling, are presented in Figure 2 and Figure 3. Thus, based on the SEM images (Figure 2), it can be noted that the CCc samples show a dendritic microstructure specific to cast alloys, but also some non-metallic inclusions and micro-shrinkages (defects specific to metallic materials obtained by casting) can also be observed. Moreover, thin scratches from the polishing process were observed on the surface.

Compared to the cast Co-Cr alloy (CCc), the one manufactured by milling (CCm) did not present dendrites, and only some very thin scratches from the milling process (in order to obtain the desired shape), and non-metallic inclusions (black colored circular formations) (Figure 3) were observed on the material surface. Considering the technique used to obtain metallic materials, some micropores may exist in both alloys.

#### 3.1.2. Chemical Composition

**Figure 4:**
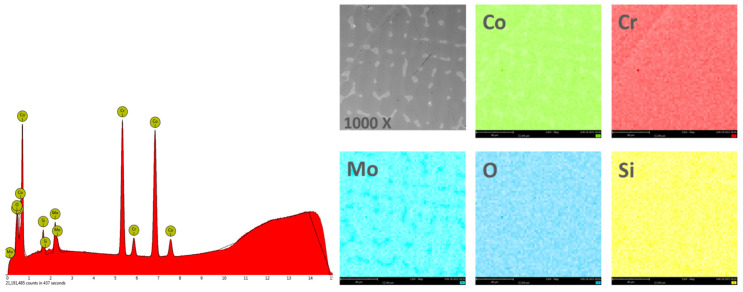
EDS spectra and elemental distribution for CCc samples.

**Figure 5:**
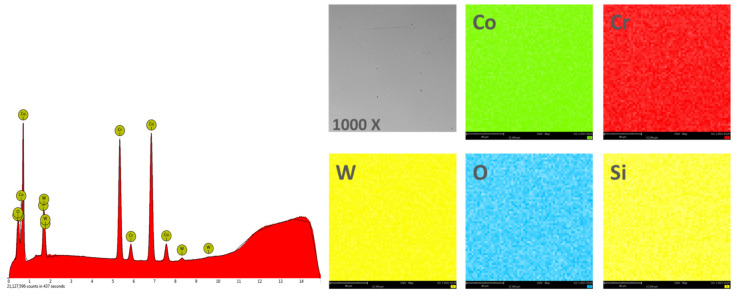
EDS spectra and elemental distribution for CCm samples.

In order to obtain the chemical composition, the EDS analysis was performed on an area of 270 × 270 μm^2^, and the obtained results are found in Table 2. Figure 4 and Figure 5 show the EDS spectra and the element distribution on the investigated area for the two Co-Cr alloys studied.

The EDS analysis indicated that the investigated Co-Cr alloys showed that the chemical composition is very close to the one specified by the manufacturer. The presence of oxygen in the chemical composition obtained by EDS is due to the oxides formed on the surface of the alloy, which protects the material against corrosive attack.

#### 3.1.3. Corrosion Resistance Testing in Artificial Saliva

Corrosion resistance was determined by the polarization resistance technique, which consists of the following steps:

- Monitoring of open circuit potential (E_OC_), for a period of 6 h;
- Tafel plots from −200 mV (vs. E_OC_) to +200 mV (vs. E_OC_), at a scanning rate of 1 mV/s;
- Plotting the linear polarization curves (of the potential dynamic curves) from −1 V (vs. E_OC_) to +1 V (vs. E_Ref_) at a scanning rate of 1 mV/s.

**Figure 6:**
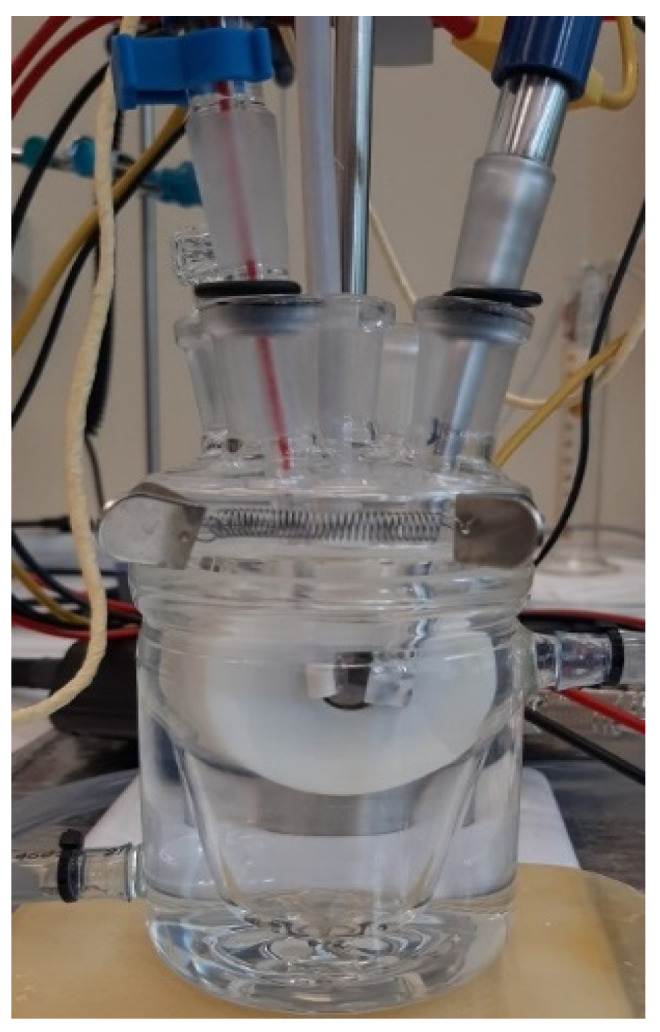
The electrochemical cell set-up used in the corrosion tests.

For corrosion tests, an area of 1 cm^2^ was exposed to the electrolyte (artificial saliva, SA). For all corrosion measurements, a three-electrode cell with the following set-up (Figure 6): sample as working electrode (WE), a platinum electrode was used as a counter electrode (CE), and saturated calomel (SCE) as reference electrode (RE), was employed.

The electrochemical cell was placed in a Faraday cage during the corrosion tests to eliminate interference due to electromagnetic fields. The samples were cleaned and degreased in acetone before the corrosion tests using an ultrasonic bath for 20 min. Subsequently, the samples were rinsed with ultrapure water (ASTM I) and dried in hot air.

The tests were performed at human body temperature (37 ± 1 °C). The tests were performed using Carter Brugirard artificial saliva as an electrolyte. 

The artificial saliva was prepared using ultra-pure water and high-purity chemicals, purchased from Sigma Aldrich (Darmstadt, Germany). The chemical composition of Carter Brugirard artificial saliva is shown in Table 3.

The experimental samples were immersed in the electrolyte having three pH values, respectively 3, 5.7, and 7.6. The pH was 7.6 immediately after preparation and was adjusted to more acidic values (5.7 and 3) by dropwise 1 M HCl solution. Table 4 presents the sample codifications used in the corrosion resistance tests.

**Figure 7:**
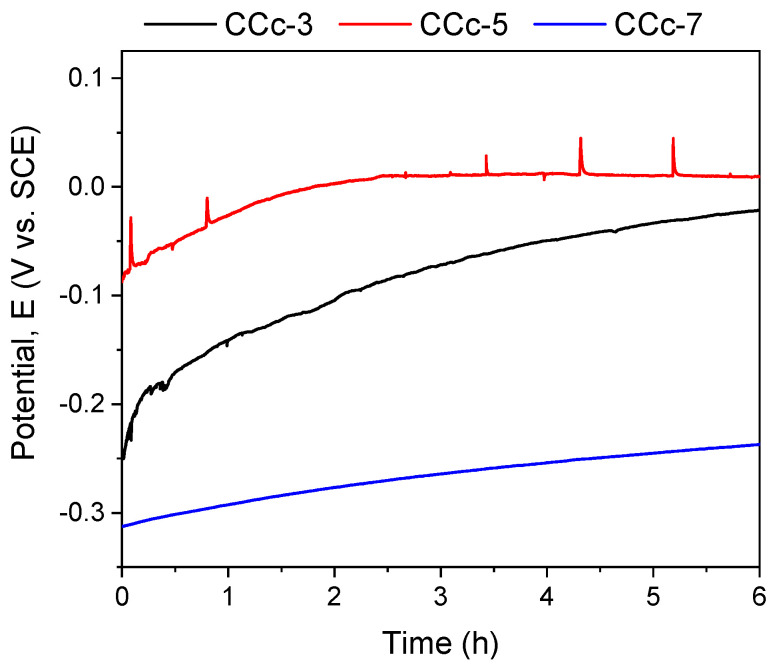
Open circuit potential evolution corresponding to the cast CoCr alloy in all testing media.

**Figure 8:**
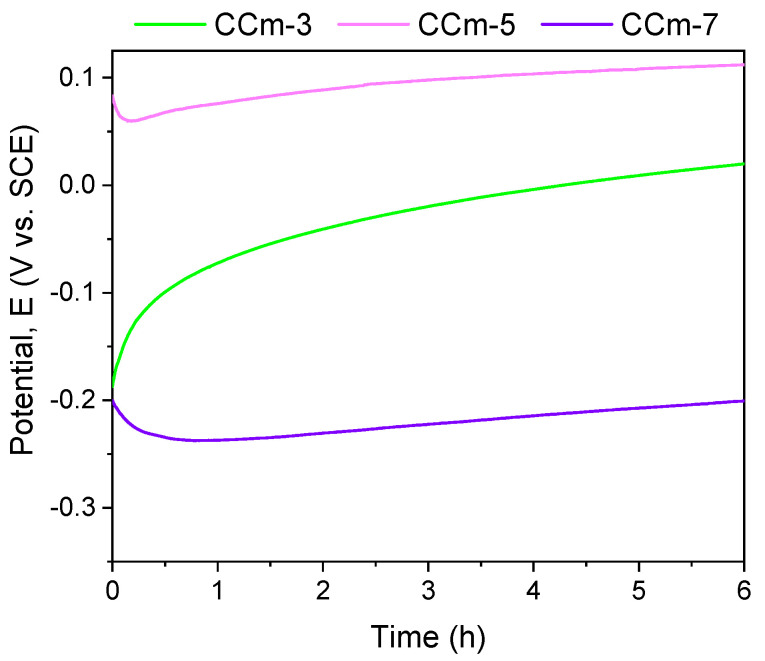
Open circuit potential evolution corresponding to the milled CoCr alloy in all testing media.

**Figure 9:**
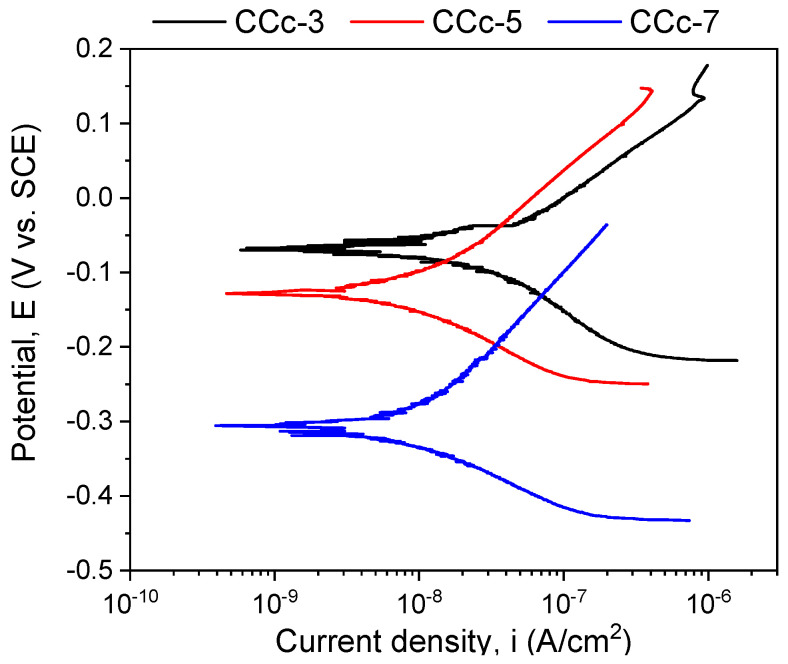
Tafel plots of cast Co-Cr alloy in all testing media.

**Figure 10:**
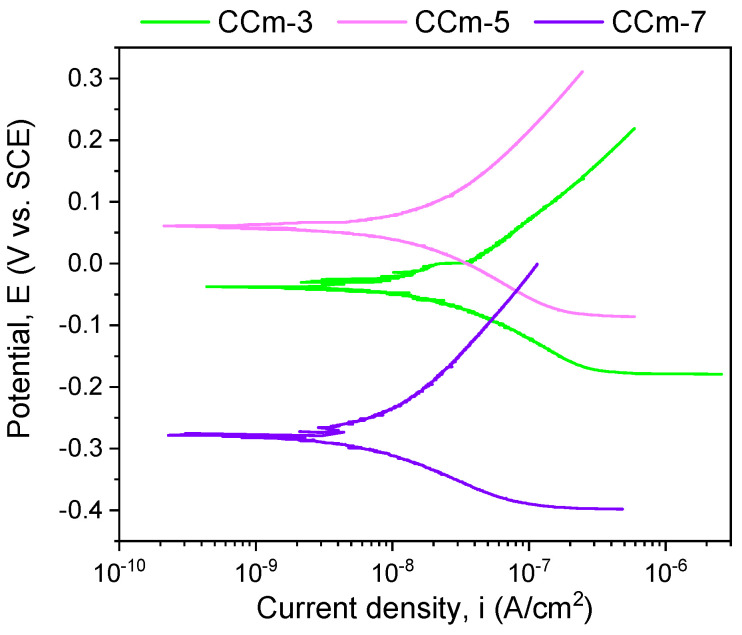
Tafel plots of milled Co-Cr alloy in all testing media.

The polarization resistance from −1 V (vs. E_OC_) to +1 V (vs. E_Ref_) was performed to highlight possible forms of corrosion that can occur at high positive potentials. Figure 7 and Figure 8 present the open circuit potential evolution (Eoc), while Figure 9 and Figure 10 present the Tafel plots of the tested samples.

The main electrochemical parameters extracted using Tafel extrapolation are presented in Table 5.

The polarization resistance (Rp) was achieved according to ASTM G59-97 (2014) using the following equation:

$$R_{p}=\frac{1}{2.3}\cdot \frac{\beta_{a}|\beta_{c}|}{\beta_{a}+\left|\beta_{c}\right|}\cdot \frac{1}{i_{\mathrm{corr}}}$$

where:

β_a_—anodic slope;
β_c_—cathodic slope;
i_corr_—corrosion current density.

The corrosion rate was calculated according to ASTM G102-89 (2015) using the formula:

$$\mathrm{CR}=K_{i}\cdot \frac{i_{\mathrm{corr}}}{\rho }\cdot \mathrm{EW}$$

where:

CR—corrosion rate;
K_i_—3.27 × 10^−3^;
ρ—material density;
i_corr_—corrosion current density;
EW—equivalent weight.

Considering the values of the electrochemical parameters, the corrosion behavior can be evaluated based on several evaluation criteria.

A more electropositive value of the open circuit potential (E_oc_) denotes a “nobler” character, and thus, a good corrosion behavior. By analyzing the allure of the curves in Figure 9, one can observe that all cast Co-Cr alloy samples have an evolution towards more electropositive values without fluctuations of the potential (except for the CCc-5 sample, but with a sudden return and without altering the evolution). This indicates that a protective and stable oxide layer is formed on its surface.

If we consider the value of the corrosion potential (E_corr_), it is known that a more electropositive corrosion potential indicates a better corrosion behavior. Thus, based on this criterion, the most electropositive potential (E_corr_) was registered in the case of the CCc-3 sample, with a value of −70 mV, followed by that of the CCc-5 sample.

In terms of corrosion current density (i_corr_), a smaller value indicates a good corrosion resistance. Thus, considering this criterion, the lowest corrosion current density was obtained for the CCc-7 sample (9.098 nA/cm^2^), followed by that of the CCc-5 sample, with a difference of only 0.387 nA/cm^2^.

It is known that a high polarization resistance highlights a better corrosion resistance of a material, and a low value of this parameter has a weaker behavior. From this point of view, it is observed that the CCc-7 sample has the highest value of this parameter (3130.56 kΩ × cm^2^), thereby demonstrating a better corrosion resistance than the other tested samples. The next value in descending order is that of sample CCc-5 (2921.24 kΩ × cm^2^).

After assessing the corrosion rate of the samples, it can be seen that the CCc-7 sample has the lowest value, of 0.096 μm/year, followed by the CCc-5 sample, at a difference of only 0.005 μm/year.

Regarding the milled Co-Cr alloy, the most electropositive open circuit potential was obtained for the CCm-5 with a value of 112 mV. By analyzing the allure of the open circuit potential, it can be observed, as in the case of cast Co-Cr alloy, that in all electrolytes, milled Co-Cr alloy tends towards more electropositive values. This can confirm the formation of a protective layer on its surface. 

The most electropositive value of the corrosion potential was obtained for the CCm-5 sample in the positive range, with a value of 54 mV. Considering the corrosion current densities, it was observed that the lowest value was registered for the CCm-7 sample (8.083 nA/cm^2^), closely followed by the CCm-5 sample (9.241 nA/cm^2^).

Among the milled Co-Cr alloy, the highest polarization resistance was obtained for the CCm-7 sample (3715.58 kΩ × cm^2^), followed by the corrosion current density by the CCm-5 sample. 

In terms of corrosion rate, the smallest value was noted for the CCm-7 sample, closely followed by the CCm-5 sample, at a very small difference of only 0.005 μm/year. 

Therefore, by analyzing all the electrochemical parameters in Table 5, it can be stated that the cast Co-Cr alloy tested in artificial saliva with three different pH values has the lowest corrosion current density, the highest polarization resistance, and the lowest corrosion rate in artificial saliva with pH = 7.6. This demonstrated enhanced resistance to corrosion than in electrolytes with a more acidic pH. 

It was also observed that the cast Co-Cr alloy tested in artificial saliva with pH = 5.7 had values close to those of the alloy tested in artificial saliva with pH = 7.6, demonstrating a fairly similar corrosion behavior. 

In the case of milled Co-Cr alloy, the same behavior was observed as in cast alloy. Moreover, it can be said that in the artificial saliva with pH = 5.7, it registered the most electropositive open circuit potential and corrosion potential values.

### 3.2. Surface Morphology after Corrosion Tests

**Figure 11:**
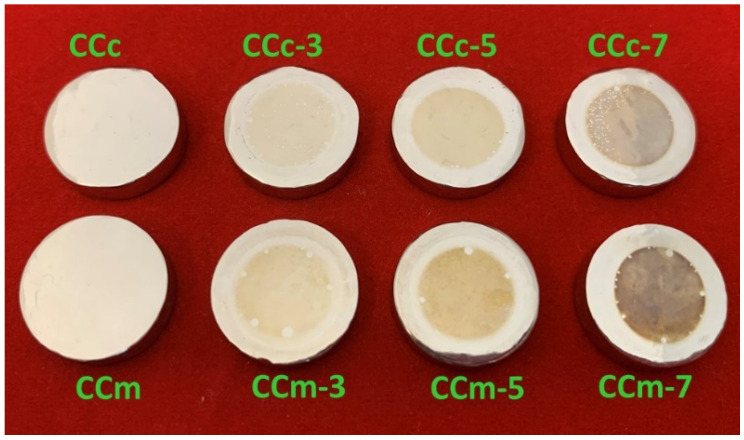
Macroscopic images of the cast and milled Co-Cr alloy after corrosion tests.

In Figure 11, macroscopic images of the cast and milled Co-Cr alloy are presented after performing the corrosion tests.

**Figure 12:**
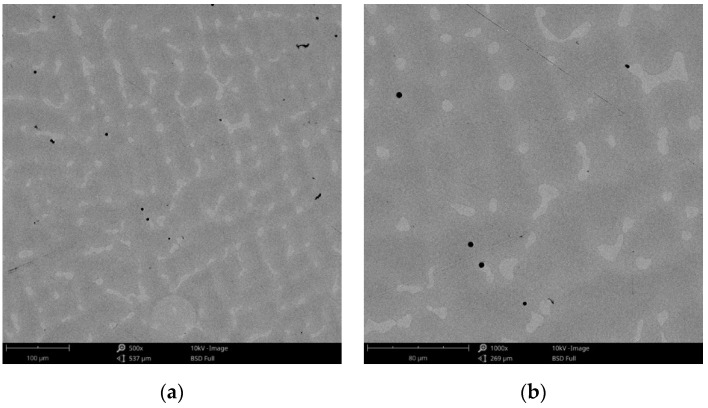
SEM images of the CCc-3 sample after corrosion tests at (**a**) 500×; (**b**) 1000×.

From the SEM images presented in Figure 12, there are no obvious forms of corrosion on the surface of all investigated samples. However, the surface exposed to the electrolyte (artificial saliva) presents some colorations after corrosion tests. In some areas, some small circular formations could be identified that have the initial color of the alloy.

These small circular areas found on the surface of the alloy were due to the hydrogen gas accumulations as gas bubbles from the electrochemical reactions.

It was also observed that by increasing the pH, the color of the exposed surface of the alloy tended towards darker shades. This may be due to the nature and thickness of the protective layer that forms on the surface of the Co-Cr alloy.

For a more detailed analysis of the sample surfaces after corrosion tests, the samples were investigated by electron microscope scanning. 

**Figure 13:**
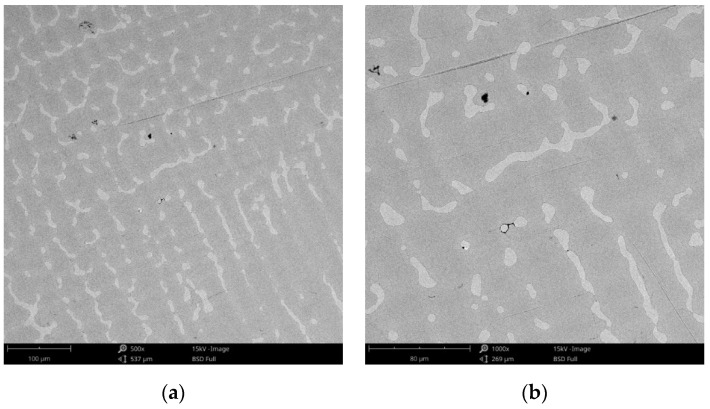
SEM images of the CCc-5 sample after corrosion tests at (**a**) 500×; (**b**) 1000×.

**Figure 14:**
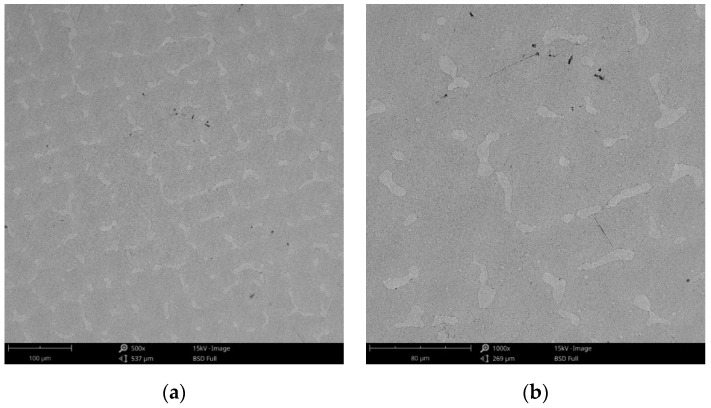
SEM images of the CCc-7 sample after corrosion tests at (**a**) 300×; (**b**) 500×.

**Figure 15:**
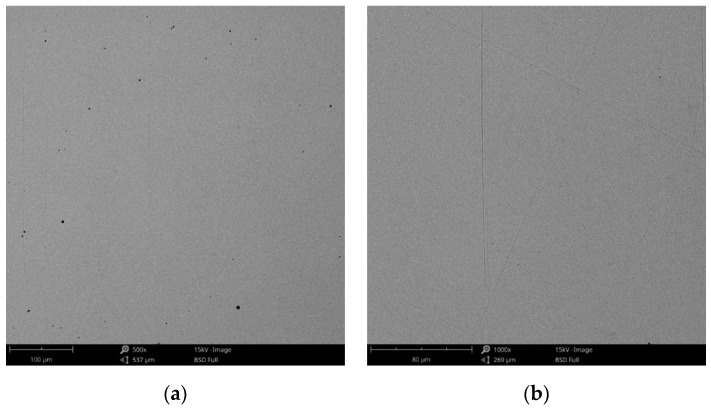
SEM images of the CCm-3 sample after corrosion tests at (**a**) 500×; (**b**) 1000×.

**Figure 16:**
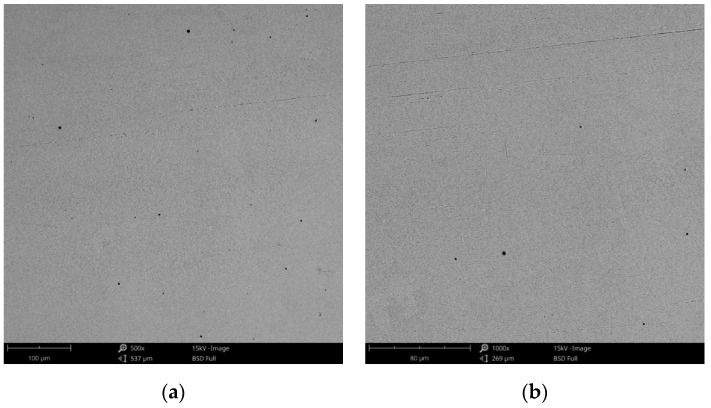
SEM images of the CCm-5 sample after corrosion tests at (**a**) 500×; (**b**) 1000×.

**Figure 17:**
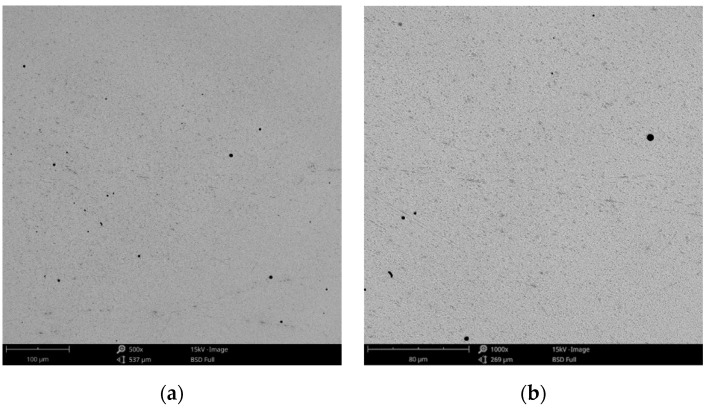
SEM images of the CCm-7 sample after corrosion tests at (**a**) 300×; (**b**) 500×.

The obtained SEM images for the cast alloy can be seen in Figure 12, Figure 13 and Figure 14 and the milled one in Figure 15, Figure 16 and Figure 17.

As the SEM images show, for the cast Co-Cr alloy tested for corrosion in artificial saliva with three pH values, no forms of corrosion were identified following the tests performed.

In milled Co-Cr alloy, no forms of corrosion were observed on its surface (Figure 15, Figure 16 and Figure 17) after corrosion tests in artificial saliva with different pH values.

Through EDS analysis, the chemical composition of the surfaces after the corrosion tests was investigated to evaluate the amount of oxygen (O), knowing that Co-Cr alloys formed a passive and stable protective oxide layer on the surface Cr_2_O_3_ is a major phase [23].

**Figure 18:**
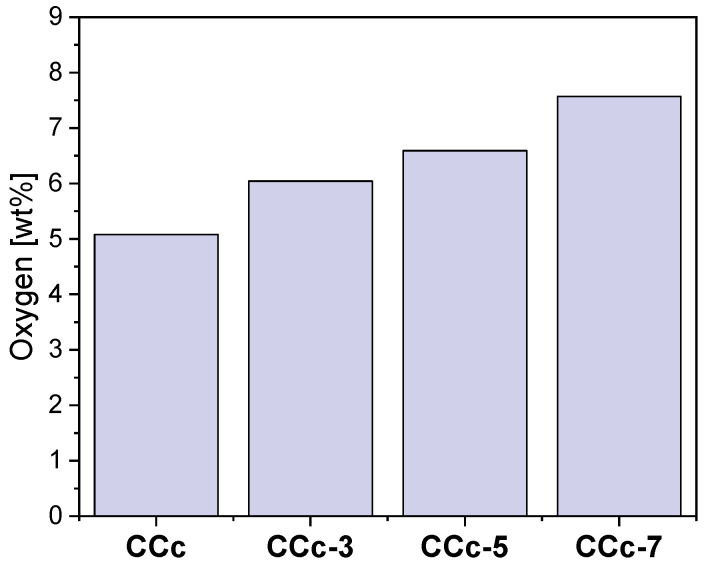
The amount of oxygen identified on the surface of the cast Co-Cr samples, before and after corrosion tests, in all electrolytes.

**Figure 19:**
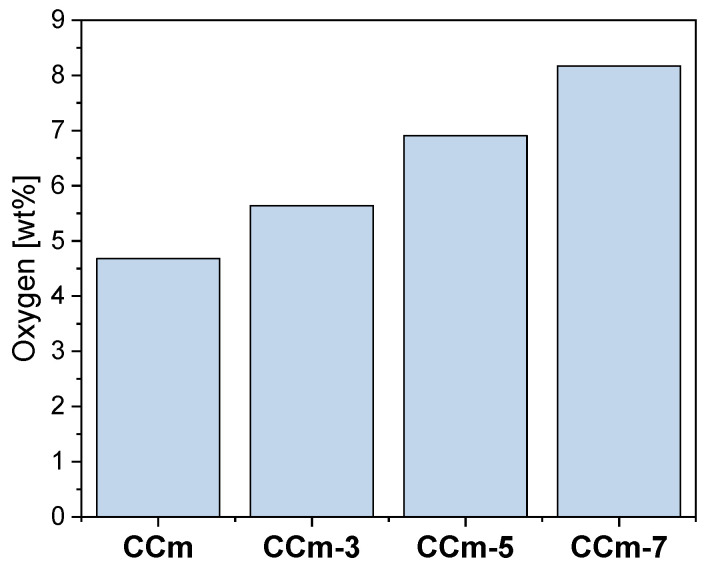
The amount of oxygen identified on the surface of the milled Co-Cr samples, before and after corrosion tests, in all electrolytes.

Thus, following the EDS investigations, the oxygen content on the surface of all samples was quantified, and the obtained results are presented in Figure 18 and Figure 19.

As seen in Figure 18 and Figure 19, oxygen increases after corrosion tests, thereby demonstrating that Co-Cr alloys form oxides on the surface as part of the protective layer.

In the case of both Co-Cr alloys, regardless of the alloying element, it could be observed that as the electrolyte’s pH value increases, the amount of oxygen also increased. This suggested that the pH value of the artificial saliva influenced the formation and the amount of the oxides on their surface.

## 4. Discussion

Nowadays, biomaterials are made of metals and alloys, ceramics, polymers, composites, and advanced materials [24,25].

Due to the high cost of noble metal alloys, base metal alloys, such as cobalt-chromium (Co-Cr), are more extensively used for the metal-ceramic restorations’ construction [26]. Nowadays, Co–Cr alloys are frequently utilized for dental restorations due to their good biocompatibility [27].

The biocompatibility of biomaterials used for dental restorations should not irritate nor induce inflammatory response or allergic reactions, respectively, nor emit toxic ions in the oro-facial system tissues [3,28].

Co-Cr alloys are frequently utilized for dental restorations, and the corrosion resistance ensures the reduction of complications in the component part of the oro-facial system. They are sufficiently chemically inert, so they are relevant in diminishing irritations, allergic reactions, and general immune system resistance [5,29]. However, because the oral environment can be aggressive due to pH variation, chloride and fluoride ions can decrease the corrosion resistance [30].

The microstructure is a very important parameter that influences the properties of the alloy and dictates its corrosion behavior. The cast samples (CCc) show a less uniform, more heterogeneous microstructure with dendrites and defects such as micropores, inclusions and micro-shrinkages. Comparatively, the microstructure of the milled alloy is more homogeneous and with fewer casting defects. It is known that single phase, fine grain, homogeneous microstructure enhances the corrosion resistance [31,32], and from this point of view, higher corrosion resistance of milled samples can be observed.

Hypersensitivity reactions can be caused by the presence of metallic ions and/or food debris, and thus the human body immune response can be altered. In the Co-Cr dental alloys used for prosthetic restorations, among the main components (Co and Cr), other elements such as Ni, Mo, Mn, etc., can also be found, which can trigger unwanted responses from the host, with either local or systemic effects [5,33,34].

As a consequence of the usage of fixed prosthodontic appliances, oral disorders, namely oral pigmentation or burning mouth syndrome, but also allergy reactions, or genotoxic and/or cytotoxic effects, might be developed [35,36,37].

After Yu J.-M. et al. [38], different manufacturing methods of metal prosthetic restorations are directly related to the microstructural characteristics of fractured surfaces of dental alloys and affect their mechanical properties. Consequently, it is imposed to comprehend the characteristics of different manufacturing methods of dental prosthetic restorations and their clinical indications.

The conditions present in the oral cavity environment (humidity, temperature, and nutrients) can favor the formation of microbial biofilms and microorganism differentiation. Nevertheless, it is known that bacteria can adhere on the surface of the dental alloys, generating metabolites as sulfides or inorganic and organic acids [39], that can influence the corrosion behavior of the alloys in long-term usage by changing oxygen concentration, salinity, and acidity of the medium [40,41]. Among the factors that can influence the corrosion resistance of an alloy, the material structure, in terms of single- or multi-phase, is of major importance because the type, concentration, and combination of the alloying elements dictate the material behavior [42]. Thus, great attention must be directed towards properly selecting the processing and manufacturing methods of the biomaterials used in dentistry.

Dental alloys should be manufactured and used with minimal harmful ion release by corrosion. Another aspect that needs to be taken into consideration is the amount and the duration of tissue contact with such elements because it can influence the biological response to the dental alloy [34]. Free ions can determine the occurrence of problems on and in the function of the prosthetic restoration and its destruction (tarnishing of metal surfaces) [43].

Because elements as Co, Cr, and Mo are considered less toxic than Ni and are released at much lower concentrations, the Co-Cr-Mo alloys are preferred, compared to Ni-Cr-based alloys, due to their enhanced biocompatibility and higher corrosion resistance [44,45].

In a study performed by Moslehifard et al. [46], it was shown that the number of metallic ions released from Co-Cr-Mo alloys was higher in buffered saline solutions than in the salivary samples, even though it remained within the physiological limit of trace elements. Haugli et al. [34] demonstrated that the cytotoxicity and inflammatory responses generated by the Co–Cr alloy could be prevented through the administration of antioxidants, therapeutic agents. Moreover, Co-Cr dental alloy induces unwanted responses in human gingival fibroblasts and osteoblasts. With saliva being particularly corrosive and playing a key role in the performance of dental alloys, many studies aimed to correlate the corrosion of the metals with the salivary pH [47].

Musa et al. [41] studied metal oxidation on Ni-Cr and Co-Cr alloys through two competing routes, oxide formation, and metal ion dissolution. They concluded that the pH value does not directly influence the thermodynamics reaction nor the nature of the oxide formed. Still, it greatly influences the relative rates of the two competing reactions.

The study of Ramırez-Ledesma et al. [48] underlines that the consequences of degradation in dental restorations caused by chloride ions fluctuation strongly correlate with their concentration in the saliva, which, in turn, depends on the oral cavity conditions such as the pH level.

Saliva is a very complex medium, which is liable for the homeostasis of the oral and digestive tract mucous membranes, contributing to protection against physicochemical aggression, thereby maintaining the integrity of the oral cavity mucosa [49]. Studies have associated the salivary pH level and volume abnormalities with GERD symptoms and laryngopharyngeal reflux disease [50].

Studies have demonstrated the importance of the content of various components in dental alloys, related to the importance of the pH of the environment [47,51,52]. Hancu et al. [53] showed that the open potential circuit values in artificial saliva with a pH of 7 are more electropositive for Co-Cr alloys than NiCr alloys, emphasizing their nobler character. The study achieved by Mercieca et al. [33] highlighted that the corrosion resistance of the Co-Cr alloy was superior to that for Ni-Cr alloy, and some changes in terms of topographical microstructure were remarked for Ni-Cr alloy after exposure to artificial saliva. Therefore, a better knowledge of the base metal alloys’ properties used in prosthetic dental restorations is necessary [3,5,22,54].

The clinical significance of this research is represented by the differentiated results between the experimental samples of the cast and milled Co-Cr dental alloys in artificial saliva at different pH values. Even though according to the obtained results, it was observed that in artificial saliva with a more acidic pH value, the investigated Co-Cr alloy, regardless of the manufacturing technique adopted (cast or milled), presented a poorer corrosion resistance. It is worth mentioning that an enhancement of the corrosion behavior was noted for the milled Co-Cr alloy, compared to cast Co-Cr alloy.

## 5. Conclusions

The conclusions that can be summarized by following this study regarding the evaluation of the corrosion behavior in artificial saliva with different pH values (3, 5.7, and 7.6) of two commercial Co-Cr alloys used for manufacturing of prosthetic restorations through two different technologies are:

1. Commercial Co-Cr alloys have the chemical composition specified by the manufacturer, and only small differences were noted;
2. All the constitutive elements of both alloys are uniformly distributed in their mass;
3. From the corrosion tests, the following was noted:
  - Both alloys presented the lowest corrosion current density, the highest polarization resistance, and the lowest corrosion rate, and thus, suggesting a better corrosion behavior in artificial saliva with the highest pH (7.6);
  - A similar tendency, in terms of electrochemical values, were found for both alloys in the case of the corrosion tests performed at pH values of 7.6 and 5.7;
  - The lowest corrosion rates values related to the pH of artificial saliva were obtained for the milled Co-Cr alloy (Co-Cr alloyed with W);
  - The cast Co-Cr alloy (Co-Cr alloyed with Mo) had smaller but very close values in terms of electrochemical parameters, indicating a poorer corrosion resistance than the milled alloy exhibited (W-alloyed Co-Cr alloy).
4. Due to a more homogeneous microstructure and fewer casting defects, the milled Co-Cr alloy exhibits a better corrosion resistance;
5. After the corrosion tests, it was noted that the surface of the samples exposed to the electrolyte is tinted/colored, and by increasing the pH of the electrolyte, the color of the exposed surface turns towards darker shades, suggesting a dependency between the thickness of the oxide film and the ph value of the electrolyte;
6. SEM investigations of the surfaces of the Co-Cr alloy after the corrosion tests revealed no forms of corrosion, indicating that the oxide layer formed on the surface is stable;
7. In the case of both Co-Cr alloys, it is observed that as the pH value of the electrolyte increases, the amount of oxygen also increases, suggesting that the pH value of the test solution influences the formation of these oxides on their surface and that with the increase in pH also the number of oxides formed increases.

After the electrochemical tests, it can be stated that both cast Co-Cr alloyed with Mo and milled Co-Cr alloyed with W has a better corrosion behavior in the saliva with higher pH values regardless of the alloying element. However, considering that the milled Co-Cr alloy presented the lowest corrosion rates values in an acidic environment, we can state that this type of alloy represents a better option for the prosthetic treatment of patients suffering from GERD.

## Figures and Tables

**Table 1:** Chemical composition specified by the manufacturer of Co-Cr alloy and sample codification.

Material Name	Manufacturing Technique	Codification	Chemical Composition [wt. %]					
			Co	Cr	Mo	Si	W	Impurities
Biomate-K Special (Simex, Italy)	Casting	CCc	60.5	31.5	5.0	2.5	-	-
Starbond Esy Disc (Scheftner Dental Alloys, Germany)	Milling	CCm	61.0	27.5	-	1.6	8.5	<1.0% (C, Mn, Fe)

**Table 2:** Chemical composition obtained by EDS of the Co-Cr alloys.

CoCr Alloy	Chemical Composition (%wt)						
	Co	Cr	Mo	O	Si	W	TOTAL
CCc	58.12	27.94	7.08	5.08	1.78	0.00	100.00
CCm	59.66	25.22	0.00	4.68	3.03	7.41	100.00

**Table 3:** Chemical composition of Carter Brugirard artificial saliva.

Substance	Quantity/L (g)
Na_2_HPO_4_	0.19
NaCl	0.7
KSCN	0.33
KH_2_PO_4_	0.26
NaHCO_3_	1.5
Ureea	1.3

**Table 4:** Coding of experimental samples from corrosion tests.

Material	Artificial Saliva pH Value	Codification
CCc (Cast alloy)	3	CCc-3
	5.7	CCc-5
	7.6	CCc-7
CCm (Milled alloy)	3	CCm-3
	5.7	CCm-5
	7.6	CCm-7

**Table 5:** The main electrochemical parameters of the investigated alloys.

Sample	E_oc_ (mV)	E_corr_ (mV)	i_corr_ (nA/cm^2^)	β_c_ (mV)	β_a_ (mV)	R_p_ (kΩ × cm^2^)	CR (μm/an)
CCc-3	−21	−70	45.155	178.84	170.94	841.56	0.481
CCc-5	9	−131	9.485	104.3	163.83	2921.24	0.101
CCc-7	−237	−311	9.098	97.34	200.28	3130.56	0.096
CCm-3	20	−35	23.805	132.67	152.57	1296.15	0.229
CCm-5	112	54	9.241	123.32	190.56	3524.57	0.089
CCm-7	−200	−278	8.083	96.58	242.50	3715.58	0.084

E_OC_—Open circuit potential; E_corr_—Corrosion potential; i_corr_—Corrosion current density; Rp—Polarization resistance, CR—Corrosion rate.
